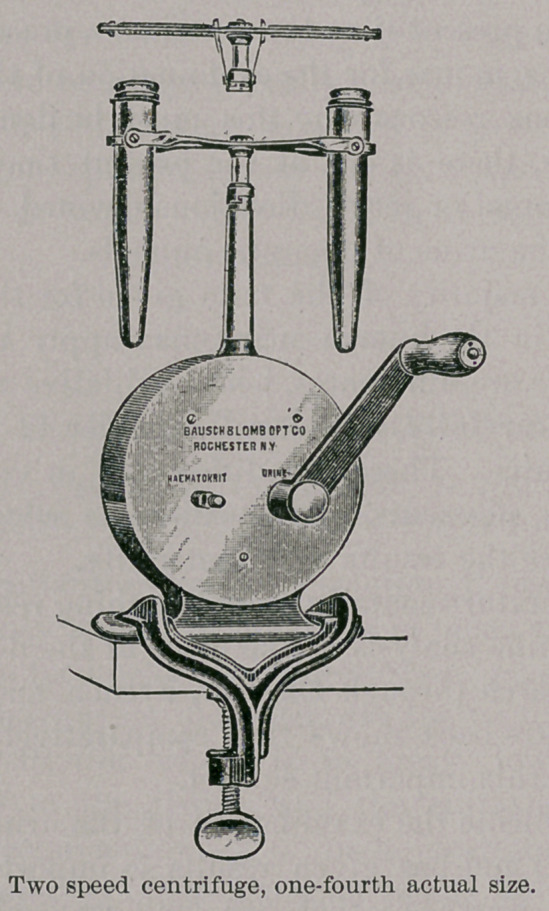# Urinary Analysis in Veterinary Practice1Read at the Thirty-seventh Annual Meeting of the American Veterinary Medical Association, Detroit, September, 1900.

**Published:** 1900-10

**Authors:** Pierre A. Fish

**Affiliations:** New York State Veterinary College, Ithaca, N. Y.


### URINARY ANALYSIS IN VETERINARY PRACTICE [1]

BY PIERRE A. FISH, D.Sc., D.V.M.,
NEW YORK STATE VETERINARY COLLEGE, ITHACA, N. Y.

THE urine essentially represents a solution of tissue constituents; certain of these may appear in the urine in much the same form as they appear in the food before it is digested—*e. g.*, sodium chloride. Other urinary constituents, however, are the result of active tissue metabolism, the effect of which is to break down the nutritive material brought to the tissues and the rearrangement of these products in new combination—*e. g.*, urea, creatinin, etc.

There is, perhaps, no other secretion in the animal body which varies so much in its composition as the urine. The amount of the constituents of a perfectly normal urine may, therefore, change within relatively wide limits without having any special significance. The analysis of urine taken from a healthy individual at different periods does not give exactly identical results. Such

[1] Read at the Thirty-seventh Annual Meeting of the American Veterinary Medical Association, Detroit, September, 1900.

variations are dependent upon diet, temperature, exercise, etc., and are purely physiological in their nature.

Abnormal urine may result from the presence of abnormal constituents, as albumin, sugar, and bile, or from the presence of one or more of the normal constituents in abnormal amount.

The urine serves as an index to tissue activity. It is the key which opens the door to the tissues and reveals their purposes.

Veterinary medicine, in its beginning, was largely dependent upon human medicine ; and while there is a broad similarity in these two branches, and, in general, the animal tissues react to certain drugs similarly to those of human tissues, there are some exceptions in which each species reacts in a manner peculiar to itself, and the reason for this is to be found in purely physiological conditions. So in the meagre analyses of the urine of the domestic animals at the present time the veterinary practitioner can only follow the outline in use for the examination of the human urine. There are obvious reasons why this must be done, for, so far as the writer knows, there is not at the present time in the English language any manual or set of directions devoted especially to the examination of the urine of domestic animals.

Although the majority of the tests given for the recognition of the constituents in the human urine may apply as well to that of the animals, there are differences, both qualitative and quantitative, which must be carefully considered in order to obtain anything like acurate results There are differences in chemical reaction, odor, consistency, pigments, and constituents which may in many instances influence the results very materially.

Veterinary literature contains almost nothing relative to a careful and purely scientific analysis of the urine of the domestic animals. A painstaking search through English, French, and German literature for some years back shows that comparatively little attention has been paid to this important subject.

In human medicine the examination of the urine has become so firmly established and has given results so important for purposes of diagnosis and prognosis that one may reasonably inquire why these undoubted benefits may not be utilized in veterinary practice. It is evident, in the majority of cases, that the veterinary practitioner has not had the necessary experience ; that he does not possess the apparatus nor the time requisite for an exhaustive or strictly scientific analysis. A mere qualitative examination, although useful for the detection of abnormal constituents, is not always sufficient. A quantitative examination has hitherto been out of the question, because, aside from the skill and apparatus necessary, a number of days would be required for a complete analysis of all of the urinary constituents.

Within a comparatively recent time new methods and apparatus have been introduced which find as ready an application in veterinary as in human usefulness. I refer especially to the centrifuge, which may now be obtained at a moderate cost, and by means of which very approximate quantitative results may be obtained in a minimum of time. For the estimation of the chlorides, sulphates, phosphates, uric acid, albumin, and urinary deposits the centrifuge is especially useful. With this apparatus but little time is required, and the information necessary for prognostic, diagnostic, or clinical purposes is readily obtained.

Two speed centrifuge, one-fourth actual size.

In veterinary practice it is seldom necessary to examine the urine of any but the horse, and what follows is therefore understood to apply to this genus.

The urine may be obtained in various ways. Sometimes it is sufficient merely to pass the hand into the rectum and press gently upon the bladder ; the catheter may be used (more easily in the female than in the male) ; or a urinal made of rubber or leather may be strapped to the horse in such a way that all of the urine may be caught.

*Quantity.* The normal quantity of urine passed by the horse in twenty-four hours is from 3000 c.c. to 6000 c.c. (three to six quarts).

*Color.* The normal color of the urine is due to pigments derived from the coloring matter of the bile. In the horse it is of a yellowish color when first passed, but turning to a deep brown after the urine has been allowed to stand for a time, or after it has been filtered.

*Transparency.* The urine of the horse is normally more or less turbid when it is passed, and it is especially so toward the end of micturition. The turbidity is due to the presence of earthy salts, chiefly the carbonates. The longer the urine remains in the bladder the more turbid does it become. The turbidity is diminished after the ingestion of large quantities of water. In other animals the urine is normally clear when it is passed, and any turbidity at that time indicates abnormal conditions. On the other hand, a clear, limpid urine in the horse is generally regarded as pathological, indicating polyuria and often showing an acid reaction.

*Consistency.* In the horse the urine is very viscid on account of the contained mucus and sometimes of epithelial débris. It filters very slowly. Urine taken directly from the ureter or the pelvis of the kidney is still more viscid, having a consistency very similar to that of egg-albumin and a high specific gravity. This would seem to indicate that the urine becomes more fluid after it has reached the bladder.

*Reaction.* In the horse the reaction is normally alkaline, and is due to the presence of the carbonates or bicarbonates of lime or potassium. A vegetable diet always influences the reaction in favor of alkalinity. It would appear that the salts present in the plants undergo oxidation to form organic acids, and these in turn are transformed into carbonates, causing alkalinity of the urine. In case of fasting or starvation, the urine of the herbivora becomes acid, which is accounted for by the fact that they are practically carnivora for the time being, and living upon their own tissue.

*Specific Gravity.* Normally the specific gravity ranges from 1020 to 1050, the average being 1035. It may fall as low as 1005 to 1010 in chronic interstitial nephritis, or rise to 1050 or 1060 in diabetes.

*Water.* The water of the urine is derived mostly from the food and drink, and varies in amount according to the activity of the skin.

*Chlorides.* The chlorides are the most abundant of any of the inorganic constituents, and are derived chiefly from the food. They are of importance clinically in that they are diminished or sometimes entirely absent in those diseases where effusions or exudates occur—*e. g.*, pneumonia. Their reappearance or increase is a favorable sign. The chlorides are also decreased in acute fevers and in some chronic diseases. They may be increased physiologically after the ingestion of salt foods and much water, and during pregnancy.

*Sulphates.* The sulphates are formed from the metabolism of the proteids in the body, and are known as ordinary sulphates. Another form known as ethereal sulphates also exists, and is present in greater amount than the former. The ethereal sulphates are formed by the combination of sulphuric acid with organic radicles, such as phenol, skatol, etc., which originate from the putrefactive processes going on in the intestine. They are of importance clinically, as they indicate any derangement of the proper performance of the digestive processes.

*Phosphates.* It has been claimed that phosphates do not normally occur in the urine of the horse. It is now generally held that they are present, but in small amount. In the urine of the omnivora and carnivora the phosphates abound. The food of the herbivora is rich in phosphoric acid, and that it does not appear in the urine except in such slight amounts is due to the fact that it forms salts in the intestines, with inorganic bases taken in with the food, and these salts are largely eliminated with the feces instead of in the urine as in the omnivora and carnivora. The phosphates exist in two forms—earthy and alkaline—the latter being more abundant. Physiologically the phosphates may be increased in the urine of the horse after a large feed of oats, bran, oil-cake, etc. Pathologically they are increased in diseases affecting the bones, as rhachitis, osteomalacia, osteoporosis, and during the active period in such bone diseases as spavin, ringbone, splint, etc. They are also increased in rheumatism and diseases of the nervous system. Clinically it is of considerable importance to examine for the amount of phosphates present.

*Urea.* This is the most important of the organic constituents, and represents about one-half of the total solids in the urine. It is the most important product of the decomposition of the proteids in the food. It is increased physiolgically by a proteid diet, exercise, and muscular vigor, and by drinking much water. It is decreased physiologically by fasting, non-nitrogenous food, and reduction of water in the diet. Pathologically urea is increased in all acute fevers, dyspnœa, diabetes, and phosphorus-poisoning. It is decreased in uræmia and in many chronic diseases. In general, any disease which interferes with the activity of the liver decreases the urea. Disease affecting the uriniferous tubules causes an appropriation of the urea from the blood and passes it on into the urine. Some medicines which increase the amount of urea are: common salt, colchicum, atropine, cantharides, ammonium chloride, coca, and salicylic acid. Some which decrease the amount of urea are: digitalis, alcohol, caffeine, the iodides of sodium and potassium, potassium bromide, arsenic, turpentine, and quinine.

*Uric Acid.* This nitrogenous constituent is present in small amount in herbivorous urine, although commonly said to be absent and its place filled by hippuric acid. The latter is present in much larger amount. In omnivora and carnivora the reverse is the case.

*Indican.* This substance is derived from indol, one of the putrefractive products formed in the intestine. It is said to exist normally in the urine of the horse to an amount twenty times as great as that found in man. Indican is of considerable clinical importance, an increase is indicative of an imperfect performance of the digestive processes. In cases of obstruction of the intestine the increase in the amount of indican is enormous.

Thus far only certain of the normal constituents of the urine have been considered. A knowledge of their presence in normal or abnormal amount in certain diseases is of the highest importance for clinical purposes. Among the important abnormal constituents found in the urine are: albumin, sugar, bile, blood, melanin, etc.

*Albumin.* The presence of this substance in the urine is regarded as pathological. According to some, there may be a physiological albuminuria for a short time, after a diet containing much albumin, or after violent exercise. The principal form of albumin present is serum-albumin; in addition there may be serum-globulin, acid-albumin, albumose, or peptone. There are special tests for distinguishing each variety, but to the practitioner the ordinary tests for albumin are sufficient, for in whatever form the albumin may be it indicates an abnormal state of affairs, and proper treatment should be administered. Fundamentally, a change in the character of the blood, a disturbance of the circulation with regard to blood-pressure, or a change in the kidney itself with respect to the epithelial cells of the uriniferous tubules, or of the glomeruli, thereby affecting transudation, account for the presence of albumin in the urine. Pathologically, albumin may appear as a result of suppression of cutaneous perspiration, in pulmonary and cardiac diseases, febrile and inflammatory diseases, lesions or prostrations of the nervous system, hydræmia and ailments that disturb the vascular tension, diseases of the genito-urinary system, and after the use of such agents as copaiba, cubebs, turpentine, and also some emetics and drastic cathartics and some anæsthetics ; also poisoning by phosphorus, iodides, and iodoform.

*Sugar.* Dextrose or glucose exists normally in the blood in the proportion of one or two parts per thousand. When a greater amount than three parts per thousand exists, the excess is excreted through the kidneys. Sugar in the urine does not necessarily point to disease of the kidney or urinary organs, but rather to the liver, indicating that its glycogenic function has been disturbed. In diabetes, the kidney in ridding itself of dextrose becomes irritated, and this irritation extends down the entire canal, thus producing a real polyuria.

Physiologically, sugar may be found in the urine during pregnancy and lactation. Pathologically, sugar is found in diabetes, in impeded respiration from pulmonary diseases, in impeded hepatic circulation due to functional and organic diseases of the liver, and in certain diseases of the nervous system ; also from the action of certain poisons, as carbon monoxide, arsenic, chloroform, and curare. The specific gravity is high and the color is usually pale, from the dilution—not diminution—of the urinary pigments. Albumin interferes with the tests, and should be removed in all cases.

*Bile.* In certain pathological conditions the elements of the bile are excreted in the urine. The pigments biliverdin or bilirubin may occur along with the bile-salts, or the bile-salts may occur alone. The urine is generally of a greenish or yellowish brown color, and froths easily. In cases of jaundice, functional or organic diseases of the liver, bile may be looked for in the urine.

*Hæmaturia,* or blood in the urine, may have its source from lesions in the kidney, as in acute nephritis, or in the ureters, bladder, or urethra. Blood may be detected by certain chemical tests, also by the use of the spectroscope and microscope.

*Melanin.* Melanin may exist in solution in the urine, but may sometimes appear in the form of a brownish or black sediment, recognizable by the microscope. An examination for this substance possesses diagnostic significance when the melanosis is beyond the reach of examination by the eye or by touch. It may disappear from the urine when the disease is arrested, or it may remain stationary. The practical significance of this condition (melanuria) is limited by the fact that the urine may contain melanin in wasting diseases, and that obtained from patients suffering from melanotic cancer may be entirely free from it. Nevertheless, as an adjunct in diagnosis, an examination for this substance is of undoubted utility.

It has not been the purpose of this paper to offer any scheme or set of directions for the analysis of herbivorous urine, but rather, by calling attention to certain constituents of the urine and the changes they may undergo qualitatively or quantitatively, to show the importance of this subject in the diagnosis and prognosis of disease, the information thus obtained oftentimes being more available and of more direct use than clinical symptoms.

## Figures and Tables

**Figure f1:**